# Editorial: Next Generation Sequencing Based Diagnostic Approaches in Clinical Oncology

**DOI:** 10.3389/fonc.2020.635555

**Published:** 2021-01-28

**Authors:** Anton Buzdin, Ira Ida Skvortsova, Xinmin Li, Ye Wang

**Affiliations:** ^1^ Shemyakin-Ovchinnikov Institute of Bioorganic Chemistry, Russian Academy of Sciences, Moscow, Russia; ^2^ World-Class Research Center “Digital Biodesign and Personalized Healthcare”, Sechenov First Moscow State Medical University, Moscow, Russia; ^3^ Translational Genome Bioinformatics Laboratory, Moscow Institute of Physics and Technology (National Research University), Moscow, Russia; ^4^ Research Department, OmicsWay Corp., Walnut, CA, United States; ^5^ Therapeutic Radiology and Oncology, Medical University of Innsbruck, Innsbruck, Austria; ^6^ Group for Experimental and Translational Radiooncology, Tyrolean Cancer Research Institute, Innsbruck, Austria; ^7^ PathoBiology Group, European Organization for Research and Treatment of Cancer (EORTC), Brussels, Belgium; ^8^ Department of Pathology & Laboratory Medicine, University of California Los Angeles (UCLA) Technology Center for Genomics & Bioinformatics, Los Angeles, CA, United States; ^9^ Clinical Laboratory, Qingdao Central Hospital, The Second Affiliated Hospital of Medical College of Qingdao University, Qingdao, China

**Keywords:** cancer diagnostics, genomics, transcriptomics, bioinformatics, biomarkers, theranostics, next generation sequencing, RNA sequencing

Next Generation Sequencing (NGS) technologies transformed cancer genetics by providing unprecedented access to big genomic and transcriptomic data (1, 2). Curation of cancer genetic profiles has led to many successful applications crosslinking mutations or polymorphisms with individual tumor response to therapies. Obviously valuable, these approaches however still cannot generate clinically actionable information for most of the cancer patients. In turn, high throughput RNA (transcriptome) analysis can be considered a rising star that may complement mutational screenings (1, 3). A combination of both approaches may be synergistic for many tasks in molecular diagnostics in oncology (4–6). Cancer mutation and expression biomarkers can not only help to set a diagnosis but also to identify appropriate personalized molecular-based treatment (7–9). Furthermore, novel bioinformatic approaches enable squeezing more and more clinically meaningful data from large genetic datasets (10).

Wang et al. reviewed current progress in using RNA sequencing (RNAseq) methods in cancer research, prognosis, and molecular diagnostics. The techniques of RNAseq have progressed rapidly from bulk RNAseq, laser-captured micro-dissected (LCM) RNAseq, and single-cell (SS) RNAseq to digital spatial RNA profiling, spatial transcriptomics, and direct *in situ* sequencing. These different technologies have their unique strengths, weaknesses, and suitable applications in the field of clinical oncology. For example, bulk RNAseq is a cost-effective mature technology that can be used for confident whole-transcriptome biomarker recovery and targeted capture of gene fusions. LCM-RNAseq interrogates tissue heterogeneity by dissecting cell type specific populations, whereas SS-RNAseq can characterize different cell types inhabiting and surrounding tumors. Zhigalova et al. applied RNAseq to characterize T-cell receptor (TCR) and immunoglobulin repertoire in an HKP1 Kras^G12D^p53^−/−^ syngeneic mouse model of lung cancer after anti-PD-1 treatment, and observed decreased TCR diversity in response to therapy. However, repertoire diversity was then restored in progressing disease but remained decreased in good responders to therapy in both CD4+ and CD8+ subsets, thus suggesting their potential usefulness as prognostic biomarkers.

Borger et al. used RNAseq and bioinformatic analysis with Oncofinder (11) for characterization of molecular processes accompanying ALPPS, a novel two-staged hepatectomy that dramatically accelerates liver regeneration and enables extensive liver tumor resection. ALPPS specific signature included activation of cell survival branch of IGF1R signaling pathway, proliferation branch of ILK Pathway, and the IL-10 Pathway, whereas the transcriptional branch of the Interferon pathway was downregulated (p < 0.05). The PAK- and ILK-associated pathways were also activated at an earlier time point, reflecting acceleration of liver regeneration (p < 0.001). Molecular therapies influencing those pathways could potentially improve the performance of ALLPS in the future.

Poddubskaya et al. used bulk transcriptomics to identify effective experimental drug combination for an advanced recurrent *ALK*-positive lung cancer patient using Oncobox algorithm (12) based on ranking of tumor-upregulated drug target genes. A 48 y.o. male patient received personalized off-label combination of crizotinib + bevacizumab + docetaxel that led to additional stabilization for 22 months. The patient survival after developing resistance to ALK inhibitor was longer for 16 months than previously reported average survival for such cases, thus evidencing effectiveness of RNA-guided prescription of cancer therapies in terms of survival and quality of life.

By experimentally *in vitro* validating meta-analysis of RNAseq data for esophageal squamous cell carcinoma (ESCC), Tang et al. identified *LINC01614* non-coding (NC) RNA that was a poor prognosis biomarker, being upregulated in ESCCs. The knockdown of *LINC01614* expression significantly inhibited the migration of ESCC cells by restricting EMT. He et al. found that another NC RNA, circular RNA *hsa_circ_0007843*, in colon cancer SW480 cells can serve as a molecular sponge by interacting with the microRNA miR-518c-5p that interacts with matrix metallopeptidase 2 (MMP2). Overexpression of *hsa_circ_0007843* promoted tumor cell growth, invasion, and migration, whereas its downregulation had opposite effects. The possible explanation for those effects is removing the miR-518c-5p inhibitory block on MMP2 translation.

Finally, Borisov and Buzdin concentrate on the phenomenon that machine learning (ML) methods that had obviously revolutionized many fields are still poorly applicable to molecular oncology. Since plausible explanation is the small size of related datasets, the authors consider using dynamic data trimming (13) to filter for more relevant and informative feature sets to apply ML for finding robust transcriptomic biomarkers using available clinically annotated datasets, e.g. (14).

Conversely, Matrone et al. reported application of cancer DNA sequencing to identify the primary origin of metastases in the patient’s thyroid gland in case of concomitant lung adenocarcinoma. The patient was diagnosed with both poorly differentiated thyroid cancer for the huge involvement of the neck and concomitant lung adenocarcinoma. Clinical features, imaging evaluation and available tumor markers couldn’t support a well-defined diagnosis, and the histologic features of the thyroid and lung biopsies confirmed the figure of two different tumors. However, the NGS analysis showed a G12C mutation in *KRAS* gene in both tissues, which is highly prevalent in lung but not thyroid cancers. Therefore, the lung origin of the disease was deduced, and the patient was addressed to the appropriate therapeutic strategy.

Yuan et al. explored *ERBB2* exon 20 insertion that is a refractory oncogenic driver, by panel NGS of 59 or 1,021 genes, in 112 lung cancer patients, including 18 patients receiving afatinib treatment. There were 66% of patients having *TP53* co-mutation, and *FOXA1* was the most prevalent co-amplified gene (in 5.5% of the cases). Patients with co-occurring *TP53* mutation showed approximately twice shorter overall survival (OS): median OS of 14.5 *versus* 30.3 months, p = 0.04. *ERBB2* exon 20 insertion also related to shorter progression-free survival (PFS): median PFS of 1.2 *versus* 4.3 months, p < 0.05.

In a subgroup of 44 cases with *ALK* gene fusions out of total 1349 lung cancer patients, Liu et al. identified *ALK* fusion partners using a customized NGS panel. The most common partner was well-known gene *EML4*, but also a new *ALK* fusion partner *HMBOX1* was identified. The copy number alterations were found in ~30% of the cases, and the most commonly amplified genes were *MDM2* and *TERT*.

Furthermore, Belardinilli et al. used target gene panel sequencing to characterize 639 formalin-fixed paraffin-embedded (FFPE) metastatic colorectal cancer (mCRC) specimens and identified blocks of significantly cooccurring mutations. This led to a novel stratification of mCRC patients in eight groups characterized by specific mutational association patterns, which was validated on a literature dataset of The Cancer Genome Atlas (TCGA) project.

By performing whole-genome sequencing, Gao et al. investigated genetic alterations in spinal schwannoma and identified mutations in *ATM, CHD4, FAT1, KMT2D, MED12, NF2*, and *SUFU* genes, and homozygous deletion was observed in *NF1, NF2*, and *CDKN2C*. Hippo signaling pathway was most significantly affected by the mutations identified. Shahid et al. used 54-gene panel for target DNA sequencing of 26 acute myeloid leukemia (AML) samples by using ultra-deep NGS with ∼5,000-coverage. Novel somatic mutations were identified, including those repeated in several genetically unrelated cancers, *i.e. STAG2* L526F and *BCORL1* A400V.

Of note, Nikitin et al. showed that Lynch Syndrome-related germline mutations (LS-mutations) in DNA mismatch repair pathway genes are statistically significantly associated with breast cancer (BC). To this end, they performed Targeted NGS of genes *MLH1, MSH2, MSH6, EPCAM*, and *PMS2* in a cohort of 492 healthy donors, 711 patients with hereditary BC, and 60 patients with sporadic BC. ~10% patients with hereditary BC had at least one germline mutation, and ~5% had predicted pathogenic mutations in these genes.

Gao et al. explored in detail the influence of sample preparation method on the results of target gene panel NGS. A 22-gene panel with 103 hotspots was used to detect mutations in paired FFPE and fresh frozen tissue specimens from 118 patients with colorectal cancer. They found that 99% of the patients one or more detectable variants, with 226 variants in FFPE and 221 in fresh tissue. Of the totally 129 individual variants identified, 96 variants were common for both FFPE and fresh biosamples, 27 were specific for FFPE, and 6 for fresh tissue specimens. These findings suggest that when there is a choice, fresh frozen specimens should be most probably considered as the preferred type of biosamples because of a number (27/129) of apparently artifact variants identified specific for the FFPE tissue samples.

Another technological aspect of NGS was investigated by Petrackova et al. who used statistical tests to assess the sequencing coverage thresholds for a robust variant calling, and published a coverage calculator. Using the sequencing error only, the recommended minimum depth of coverage was 1,650 along with a threshold of no less than 30 variant reads for a targeted clinical NGS mutation analysis. This points to an important problem of data quality standardization in clinical NGS. However, in practice this threshold can be only applied to target panel gene sequencing methods, whereas it is so far unrealistic for the whole-genome or even exome sequencing approaches.

Finally, epigenetic aspects of using NGS in oncology were considered by Jovčevska who reviewed current progress in sequencing and machine learning applications for the analysis of DNA methylation in glioblastoma.

## Author Contributions

All authors listed have made a substantial, direct, and intellectual contribution to the work and approved it for publication.

## Funding

AB contribution was supported by the Russian Science Foundation grant 18-15-00061. YW was sponsored by the National Natural Science Foundation of China (No. 81670822), and Qingdao Key Research Project (No. 19-6-1-3-nsh).

## Conflict of Interest

Author AB was employed by the company OmicsWay Corp.

The remaining authors declare that the research was conducted in the absence of any commercial or financial relationships that could be construed as a potential conflict of interest.
